# Supplementary material for: Nanostructured Lipid Carriers Enriched Hydrogels for Skin Topical Administration of Quercetin and Omega-3 Fatty Acid
Source: Pharmaceutics. 2023 Aug 3;15(8):2078. doi: 10.3390/pharmaceutics15082078 (PMC10459668; doi:10.3390/pharmaceutics15082078)
Supplement: Supplementary file 1 [file pharmaceutics-15-02078-s001.zip › pharmaceutics-2498260-supplementary.pdf]

Supplementary materials

Nanostructured Lipid Carriers Enriched Hydrogels for Skin Topical Administration of Quercetin and Omega-3 Fatty Acid

Marlene Lúcio [1,2,*], Nicole Giannino [3,4], Sérgio Barreira [3], José Catita [3,5], Hugo Gonçalves [5], Artur Ribeiro [6,7], Eduarda Fernandes [1], Isabel Carvalho [6,7,8], Hugo Pinho [3], Fátima Cerqueira [3,9], Marco Biondi [4] and Carla M. Lopes [3,10,11,*]

Table S1. Pre-formulation studies of Q (0.2% *w/w*) in different solid and liquid lipids.

Solid Lipid(s)	15 minutes	30 minutes	45 minutes	60 minutes
Precirol® ATO 5	-	-	-	-
Compritol® 888 ATO	-	-	-	-
Cetyl palmitate	-	-	-	-
Gelucire® 50/13	-	+	+	+
Stearic acid	-	-	-	-
Liquid Lipid(s)				
Miglyol® 812	-	-	-	-
Capryol 90	-	-	-	-
Labrasol	+	+	+	+
Labrafac	-	-	-	-
ω_3	-	-	-	-
Lipid Mixture				
Precirol® ATO 5 + Gelucire® 50/13 + ω_3	-	-	-	-
Precirol® ATO 5 + Ómega 3	-	-	-	-
Precirol® ATO 5 + Gelucire® 50/13 + Labrasol+ ω_3	-	-	+	+

Abbreviations: Q – quercetin; ω_3 – omega 3 fatty acid. (-) represents samples with macroscopic and microscopic signs of non-solubilization of Q and (+) represents samples with macroscopic and microscopic uniform appearance:

(+) Macroscopic appearance

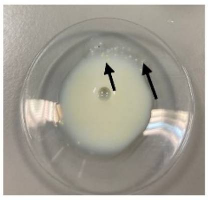

(+) Microscopic appearance

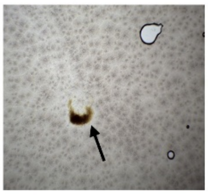

(-) Macroscopic appearance

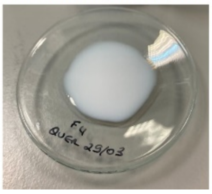

(-) Microscopic appearance

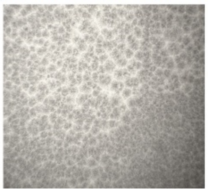

Table S2. Composition of nanostructured lipid carriers (NLCs) (*w/w*).

Components	NLC1				NLC2			
	**Placebo**	**+ Q**	**+ ω3**	**+ Q + ω3**	**Placebo**	**+ Q**	**+ ω3**	**+ Q + ω3**
**Lipid phase**								
Q	–	0.050	–	0.050	–	0.050	–	0.050
ω3	–	–	0.200	0.200	–	–	0.200	0.200
Precirol ATO® 5	4.200	4.179	4.116	4.095	4.200	4.179	4.116	4.095
Gelucire® 50/13	2.800	2.786	2.744	2.730	2.800	2.786	2.744	2.730
Labrasol®	3.000	2.985	2.940	2.925	3.000	2.985	2.940	2.925
Soy lecithin	1.500	1.500	1.500	1.500	–	–	–	–
**Aqueous phase**								
Poloxamer 407	1.500	1.500	1.500	1.500	–	–	–	–
Tween® 80	–	–	–	–	2.500	2.500	2.500	2.500
DOSS	–	–	–	–	0.500	0.500	0.500	0.500
Purified water	87.000	87.000	87.000	87.000	87.000	87.000	87.000	87.000

Abbreviations: Q – quercetin; ω3 – omega 3 fatty acid; DOSS – dioctyl sodium sulfosuccinate

Table S3. DSC thermal events of the NLCs′ lipid components and active: enthalpy (ΔH in $J.g^{-1}$) and temperature (T in °C) of the transitions.

Samples	T_{onset} (°C)	T_{peak} (°C)	T_{endset} (°C)	ΔH ($J.g^{-1}$)
**ω3**	–	–	–	–
**Labrasol®**	–	–	–	–
**Precirol ATO® 5**	38.0	52.0 (shoulder)<br>56.9 (peak)	64.9	22.0 (shoulder)<br>57.3 (peak)
**Gelucire® 50/13**	22.0	32.0 (peak 1)<br>37.1 (peak 2)<br>44.4 (peak 3)	58.0	4.4 (peak 1)<br>8.1 (peak 2)<br>14.2 (peak 3)
**Q**	116.0	129.2	139.0	61.2
**Q in the lipid melted mixture**	19.0	32.0 (peak 1)<br>55.5 (shoulder)<br>59.7 (peak 3)	63.0	16.0 (peak 1)<br>14.5 (shoulder)<br>37.7 (peak 3)
**NLC1**	23.5	49.5 (peak 1)<br>54.5 (shoulder)<br>64.0 (peak 3)	65.0	10.0 (peak 1)<br>10.5 (shoulder)<br>29.6 (peak 3)
**NLC2**	23.5	49.5 (peak 1)<br>54.0 (shoulder)<br>59.7 (peak 3)	65.0	10.0 (peak 1)<br>10.5 (shoulder)<br>37.7 (peak 3)

Table S4. Fittings, using different mathematical models, of Q release kinetics from NLC1 and NLC2 (with or without ω3) in medium mimetic of skin conditions (pH 5.6 and temperature of 32 ± 3 °C).

1st Order kinetics model	NLC	Adjusted parameters with 95% confidence interval	R^2	R^2 adjusted
$y = F_{max}(1 - e^{-kt})$	NLC1 + Q	F_{max}= 61.2<br>(56.2 – 66.2)<br>k = 1.26×10^{-3} min^{-1}<br>(1.09×10^{-3} – 1.42×10^{-3}) min^{-1}	0.992	0.991
	NLC1 + Q + ω3	F_{max}= 62.3<br>(56.4 – 68.2)<br>k = 1.45×10^{-3} min^{-1}<br>(1.20×10^{-3} – 1.71×10^{-3}) min^{-1}	0.985	0.983
	NLC2 + Q	F_{max}= 56.7<br>(50.0 – 63.4)<br>k = 7.39×10^{-4} min^{-1}<br>(6.08×10^{-4} – 8.70×10^{-4}) min^{-1}	0.995	0.994
	NLC2 + Q + ω3	F_{max}= 51.7<br>(47.9 - 55.5)<br>k = 1.07×10^{-3} min^{-1}<br>(9.43×10^{-4} – 1.20×10^{-3}) min^{-1}	0.995	0.994

F_{max} is the total amount of Q released and k is the first order release constant.

Korsmeyer–Peppas model	NLC	Adjusted parameters with 95% confidence interval	R^2	R^2 adjusted
$y = at^n$	NLC1 + Q	a = 0.355<br>(0.185 – 0.525)<br>n = 0.690<br>(0.617 – 0.762)	0.969	0.966
	NLC1 + Q + ω_3	a = 0.476<br>(0.208 – 0.745)<br>n = 0.657<br>(0.574 – 0.741)	0.960	0.956
	NLC2 + Q	a = 0.113<br>(0.070 – 0.155)<br>n = 0.800<br>(0.745 – 0.855)	0.989	0.988
	NLC2 + Q + ω_3	a = 0.229<br>(0.137 – 0.321)<br>n = 0.716<br>(0.657 – 0.776)	0.980	0.978
$y = a(t-l)^n$	NLC1 + Q	a = 1.459<br>(1.039 – 1.862)<br>n = 0.496<br>(0.454 – 0.538)<br>l = 105.4 min<br>(89.1 – 121.7) min	0.993	0.992
	NLC1 + Q + ω_3	a = 2.131<br>(1.308 – 2.953)<br>n = 0.452<br>(0.396 – 0.509)<br>l = 111.2 min<br>(92.4 – 129.9) min	0.984	0.981

NLC2 + Q	a = 0.441<br>(0.330 – 0.552)<br>n = 0.616<br>(0.581 – 0.651)<br>l = 94.3 min<br>(80.5 – 112.2) min	0.998	0.998
NLC2 + Q + ω_3	a = 0.842<br>(0.620 – 1.064)<br>n = 0.539<br>(0.502 – 0.577)<br>l = 100.6 min<br>(83.4 – 117.8) min	0.996	0.995

a is a constant of geometric and structural incorporation that considers the pharmaceutical form; n is a release representing the mechanism of diffusion of Q, being based on Fick's law, (a value of n equal to or less than 0.5 indicates a Fickian diffusion, values between 0.5 and 1 indicates a non-Fickian diffusion). l is the lag time.

Weibull model	NLC	Adjusted parameters with 95% confidence interval	R^2	R^2 adjusted
$y = F_{max}\left(1 - e^{(-at^b)}\right)$	NLC1 + Q	$a = 2.52\times10^{-4}$ ($1.97\times10^{-4} - 3.06\times10^{-4}$) $F_{max}= 51.5$ (50.8 – 52.2) $b = 1.320$ (1.281 – 1.360)	0.999	0.999
	NLC1 + Q + ω_3	$a = 1.33\times10^{-4}$ ($6.61\times10^{-6} - 2.60\times10^{-4}$) $F_{max}= 53.8$ (52.0 – 55.6) $b = 1.458$ (1.289 – 1.627)	0.995	0.994
	NLC2 + Q	$a = 1.43\times10^{-4}$ ($1.02\times10^{-4} - 1.84\times10^{-4}$) $F_{max}= 38.4$ (37.6 – 39.2) $b = 1.369$ (1.315 – 1.423)	0.999	0.999
	NLC2 + Q + ω_3	$a = 2.57\times10^{-4}$ ($1.72\times10^{-4} - 3.43\times10^{-4}$) $F_{max}= 42.0$ (40.9 – 42.9) $b = 1.293$ (1.231 – 1.354)	0.999	0.999

F_{max} is the total amount of Q released and a is a parameter that defines the time scale of the process. The parameter b characterizes the shape of the curve. For $b = 1$ it is sigmoid, for $b > 1$ is S-shaped with a curvature upwards followed by a turning point, for $b < 1$ is parabolic with an initial slope and then exponential.

Higuchi model	NLC	Adjusted parameters with 95% confidence interval	R^2	R^2 adjusted
$y = Kt^{0.5}$	NLC1 + Q	K = 1.188 (1.069 – 1.307)	0.906	0.901
	NLC1 + Q + ω_3	K = 1.334 (1.208 – 1.460)	0.917	0.913
	NLC2 + Q	K = 0.814 (0.713 – 0.916)	0.877	0.870
	NLC2 + Q + ω_3	K = 0.939 (0.852 – 1.026)	0.909	0.905

K is the Higuchi dissolution constant.

**Gallagher-Corrigan model**	**NLC**	**Adjusted parameters with 95% confidence interval**	**R^2**	**R^2 adjusted**
$$y = F_b\,(1 - e^{-k_1 t}) + (F_{max} - F_b)\left(\frac{e^{k_2 t - k_2 t_{max}}}{1 + e^{k_2 t - k_2 t_{max}}}\right)$$	NLC1 + Q	$F_b = 1.0$<br>(0 – 15.4)<br>$F_{max} = 49.4$<br>(45.9 – 52.8)<br>$t_{max} = 387.6$ min<br>(318.1 – 456.8) min<br>$k_1 = 8.16\times10^{-3}$ min^{-1}<br>($0 - 1.89\times10^{-1}$) min^{-1}<br>$k_2 = 7.30\times10^{-3}$ min^{-1}<br>($4.71\times10^{-3} - 9.89\times10^{-3}$) min^{-1}	0.981	0.975
	NLC1 + Q + ω_3	$F_b = 6.36\times10^{-4}$<br>(0 – 5.03)<br>$F_{max} = 53.0$<br>(48.8 – 56.2)<br>$t_{max} = 346.3$ min<br>(313.2 – 379.4) min<br>$k_1 = 0.281$ min^{-1}<br>(0.281 – 0.281) min^{-1}<br>$k_1 = 8.44\times10^{-3}$ min^{-1}<br>($5.72\times10^{-3} - 1.12\times10^{-2}$) min^{-1}	0.983	0.977
	NLC2 + Q	$F_b = 1.00\times10^{-2}$<br>(0.00 – 7.63)<br>$F_{max} = 36.1$<br>(34.5 – 37.7)<br>$t_{max} = 420.9$ min<br>(360.1 – 481.7) min<br>$k_1 = 8.65\times10^{-3}$ min^{-1}<br>(0.00 – 10.4) min^{-1}	0.991	0.988

	k_2 = 7.37×10^{-3} min^{-1}<br>(4.72×10^{-3} – 1.00×10^{-2}) min^{-1}		
NLC2 + Q + ω3	F_b = 7.62×10^{-4}<br>(0.00 – 3.14)<br>F_{max} = 39.5<br>(37.3 – 41.6)<br>t_{max} = 410.9 min<br>(375.4 – 446.3) min<br>k_1 = 0.668 min^{-1}<br>(0.668 – 0.668) min^{-1}<br>k_2 = 6.79×10^{-3} min^{-1}<br>(4.78×10^{-3} – 8.80×10^{-3}) min^{-1}	0.984	0.979

F_b is the amount of Q released directly from surface of the system (initial burst), F_{max} is the amount of Q released during the process, t_{max} is the time (in hours) in which occurs the release of maximum amount of Q directly from the surface of system (after burst), k_1 and k_2 are constants of release (h^{-1}) of first and second phase, respectively.

Table S5. Statistical analysis applied to the antioxidant effect results. Comparisons were performed using one-way ANOVA with the Tukey's multiple comparison test (*p-value* <0.05).

**Paired observations**	**[Antioxidant] mM**	**[MDA] µM**
NLC1 Placebo vs NLC1 + Q	**	***
NLC1 Placebo vs NLC1 + ω_3	ns	***
NLC1 Placebo vs NLC1 + Q + ω_3	***	ns
NLC1 + Q vs NLC1 + Q + ω_3	***	***
NLC1 Placebo vs NLC2 Placebo	ns	ns
NLC1 + Q vs NLC2 + Q	***	***
NLC1 + ω_3 vs NLC2 + ω_3	ns	***
NLC1 + Q + ω_3 vs NLC2 + Q + ω_3	***	***
NLC2 Placebo vs NLC2 + Q	***	***
NLC2 Placebo vs NLC2 + ω_3	ns	***
NLC2 Placebo vs NLC2 + Q + ω_3	***	***
NLC2 + Q vs NLC2 + Q + ω_3	ns	ns
Q vs ω_3	***	***
Q vs Q + ω_3	ns	***
ω_3 vs Q + ω_3	***	***
NLC1 + Q vs Q	***	***
NLC2 + Q vs Q	***	ns
NLC1 + ω_3 vs ω_3	ns	*
NLC2 + ω_3 vs ω_3	ns	***
NLC1 + Q + ω_3 vs Q + ω_3	ns	***
NLC2 + Q + ω_3 vs Q + ω_3	***	***

*** indicates a value of $p < 0.001$, ** indicates a value of $p < 0.01$ and * indicates a value of $p < 0.05$ which were considered statistically significant. ns indicates no statistical significance.

Table S6. [MDA] in mg/Kg

NLC1 Placebo	5.5
NLC1 + Q	2.9
NLC1 + ω_3	3.7
NLC1 + Q + ω_3	6.0
NLC2 Placebo	5.7
NLC2 + Q	0.0
NLC2 + ω_3	0.3
NLC2 + Q + ω_3	0.3
Q	0.0
ω_3	4.9
Q + ω_3	12.4

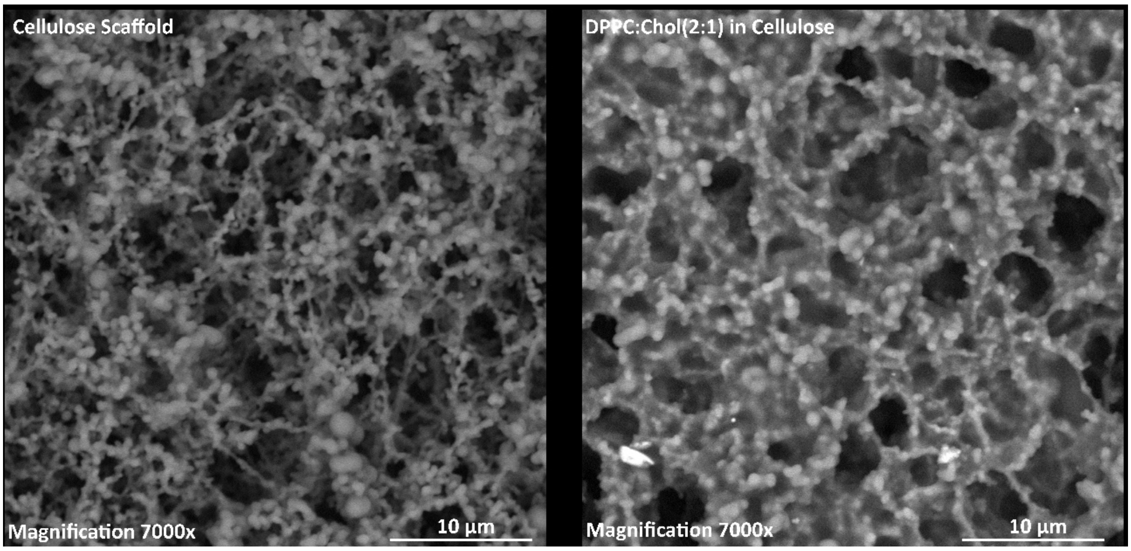

Figure S1. Morphological characterization of a mimetic *stratum corneum* model, demonstrating the lipid coverage of the scaffold while maintaining the integrity of its pores at the same magnification.

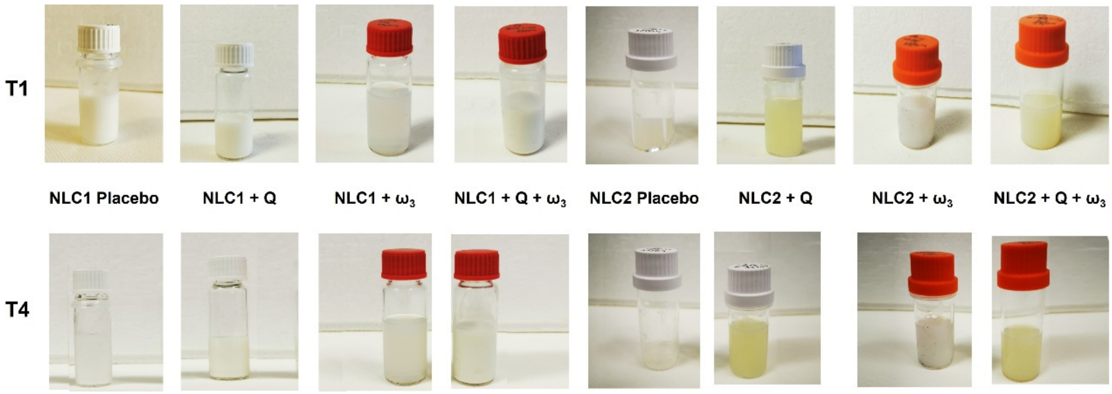

Figure S2. Macroscopic appearance of the NLCs' formulations in shelf conditions (4 °C temperature with no light exposure) on the preparation day (T1) and after four weeks (T4).

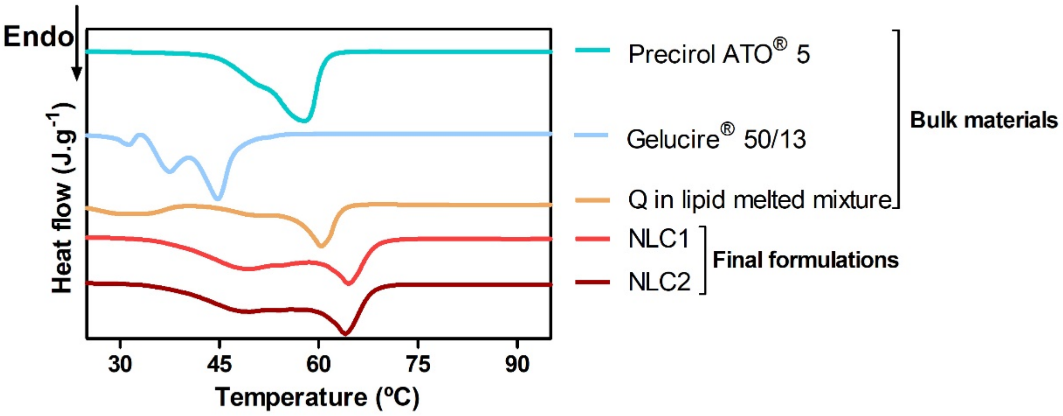

Figure S3. DSC thermograms of the NLCs components and Q in the lipid melted mixture (physical mixture of bulk materials) and of the final formulations NLC1 and NLC 2, performed after evaporation of the aqueous phase.

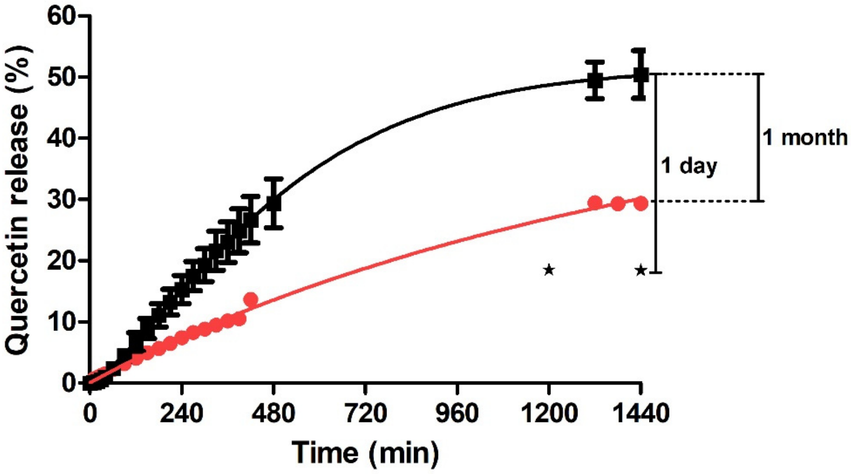

Figure S4. Quercetin release over time. Black squares – quercetin quantified immediately after its release from NLC1+Q freshly produced (T1). Red circles - quercetin quantified immediately after its release from NLC1+Q stored 1 month in the fridge (T4). Black stars - quercetin quantified 1 day after its release from NLC1+Q freshly produced (T1).

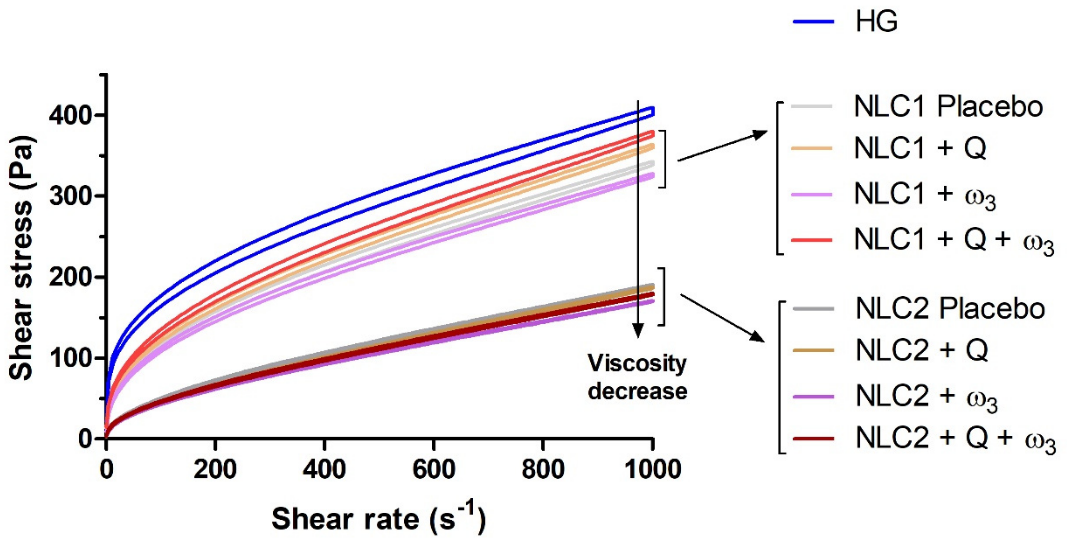

Figure S5. Rheological analysis of NLCs enriched HGs

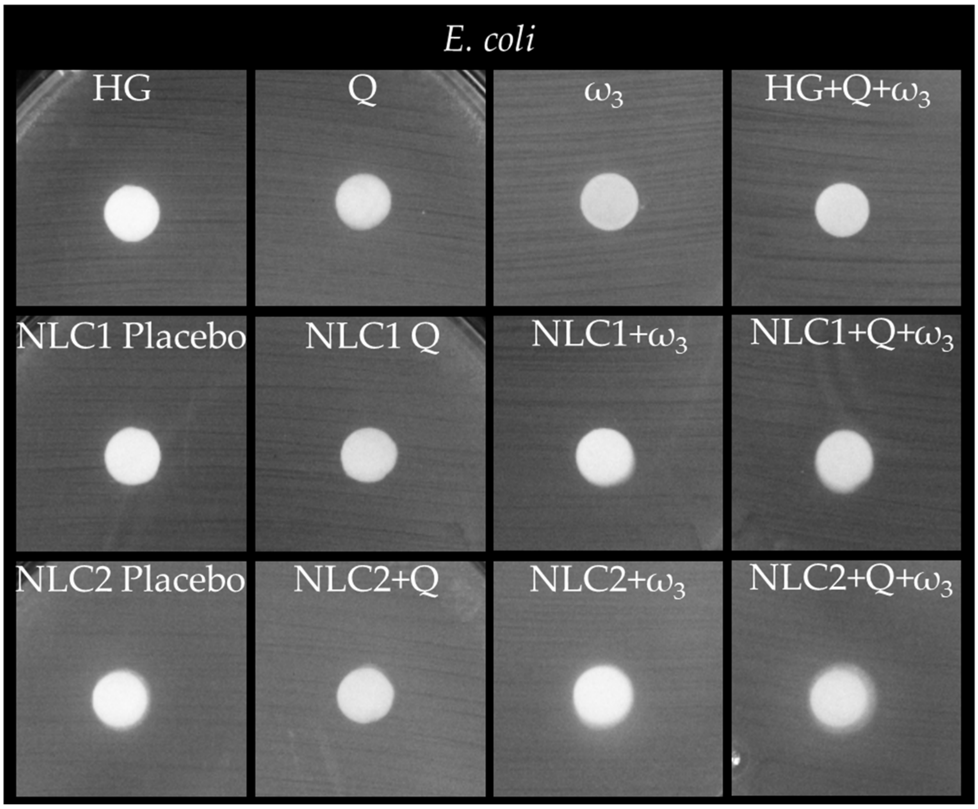

Figure S6. Evaluation of antibacterial activity of NLCs enriched hydrogels by the disk agar diffusion method against *E. coli*.

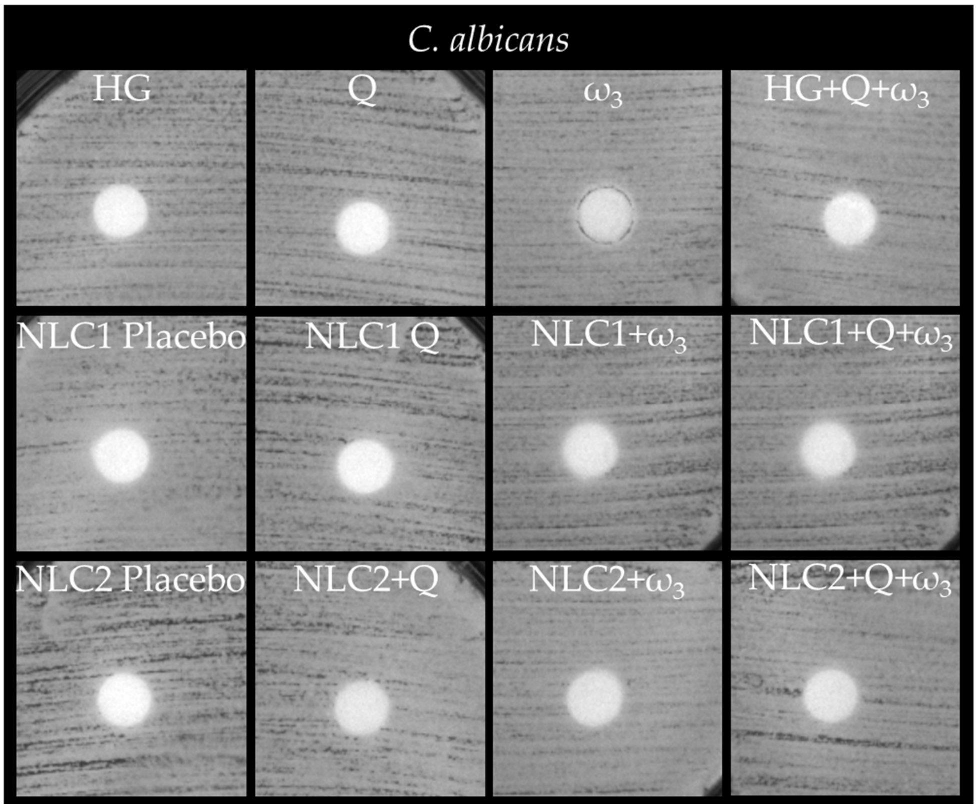

Figure S7. Evaluation of antifungal activity of NLCs enriched hydrogels by the disk agar diffusion method against *C. albicans*.

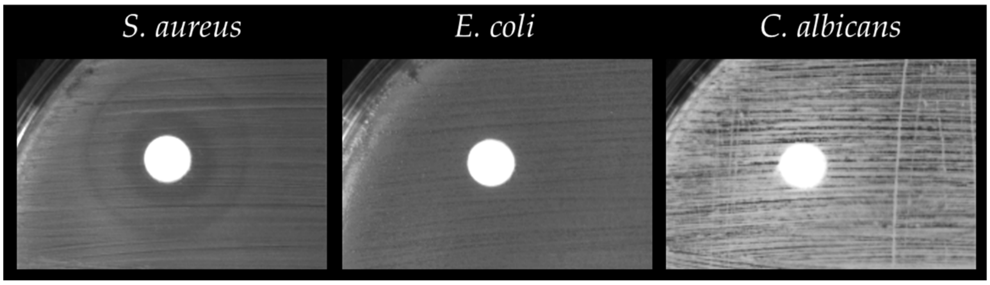

Figure S8. Evaluation of antibacterial and antifungal activities of the combination of Tween® 80 and DOSS by the disk agar diffusion method.
